# Hepatitis B Viral DNA Among HBs Antigen Negative Healthy Blood Donors

**DOI:** 10.5812/hepatmon.6590

**Published:** 2013-03-17

**Authors:** Maryam Vaezjalali, Shabnam Rashidpour, Hanieh Rezaee, Bashir Hajibeigi, Majid Zeidi, Latif Gachkar, Shadi Aghamohamad, Ronak Najafi, Hossein Goudarzi

**Affiliations:** 1 Infectious Diseases and Tropical Medicine Research Center, Shahid Beheshti University of Medical Sciences, Tehran, IR Iran; 2 Department of Microbiology, Faculty of Medicine, Shahid Beheshti University of Medical Sciences, Tehran, IR Iran; 3 Department of Immunology, Faculty of Medicine, Shahid Beheshti University of Medical Sciences, Tehran, IR Iran; 4 Blood Transfusion Research Center, High Institute for Research and Education in Transfusion Medicine, Tehran, IR Iran

**Keywords:** Hepatitis B virus, Blood Donors, Polymerase Chain Reaction, Hepatitis B Surface Antigens

## Abstract

**Background:**

Presence of occult hepatitis B infection (OBI) renders HBs antigen (HBsAg) undetectable by ELISA. Therefore it is valuable to evaluate the frequency of OBI among healthy blood donors to improve and perhaps change the strategies of blood screening to reduce the risk of HBV transmission.

**Objectives:**

The aim of this study was to determine the presence of HBcAb and HBV DNA among Iranian HBsAg negative healthy blood donors who donated their blood to the Tehran Blood Transfusion Center during 2011.

**Patients and Methods:**

1000 serum specimens negative for HBsAg, HCV antibody and HIV antibody were collected from healthy blood donors and tested for HBcAb. Presence of hepatitis B viral DNA was checked in HBcAb positive samples by nested PCR with two sets of primers to amplify part of HBV S gene.

**Results:**

There were 64 women and 936 men in the population under study. The mean ± SD age of the donors was 38 ± 11 years. 80 out of 1000 samples (8%) were found to be positive for HBcAb. HBV DNA was detected in 50% of HBcAb positive specimens. The mean ± SD age of donors without HBV DNA was 37.7 ± 10.5 years and for donors with HBV DNA was 40.9 ± 11.2 years (P = 0.05).

**Conclusions:**

OBI was prevalent among 50% of HBcAb positive healthy blood donors. The frequency of positive HBcAb among healthy HBsAg negative blood donors was comparable to previous studies reported from Iran. On the other hand, the frequency of HBV DNA in HBsAg negative blood donors was higher than previous reports.

## 1. Background

Infection with hepatitis B virus (HBV) may lead to acute, chronic and sometimes occult infection. HBV has different endemicity rates in the world. In the Middle East, some countries experience high infection rates for HBV infection (Oman and Yemen) while some other countries (Iran and Pakistan) perceived low endemicity for HBV infection (1). The prevalence of occult hepatitis B infection (OBI) is variable depending on different endemicity of HBV infection in the populations where the relevant study has been done, on different samples (liver specimen or serum) to detect HBV DNA, and on different assays that have been utilized in the study (2). Basically, OBI is characterized by the presence of HBV DNA in the serum of HBsAg negative individuals with serological markers of previous infection such as HBcAb (3). Therefore when using available ELISA tests, there is still a potential risk for transmission of HBV infection in the phase of OBI through blood transfusion. This potential can be avoided if more sensitive tests and preferentially molecular tests are done in endemic regions of this infection. Many recipients of blood products have chronic congenital or acquired diseases and most of them are immunosuppressive to some extent. HBV transmission through transfusion among these hosts may result in severe type of hepatitis B disease in comparison with a normal host. On the other hand, researchers from India (4) and Taiwan (5) have identified OBI as the major cause of HBV transmission through transfusion. Interestingly in both of these countries the incidence of post transfusion hepatitis B is still anything but negligible. HBsAg ELISA test is the only test for donor screening in many countries. HBcAb with consistent nucleic acid tests (NAT) may be a helpful test to diagnose possible OBI (6). Some researchers believe that in endemic regions where the prevalence of OBI is a major concern, a more appropriate method like HBV NAT is needed to ensure the safety of blood products (7). The cost of NAT assays for HBV detection together with the economical statues of HBV endemic countries are obstacles to employ nucleic assays for blood screening in these countries (8). It is extremely valuable to determine the prevalence of OBI among healthy blood donors to estimate the chance of HBV transmission through blood transfusion and then assess the need to improve and even change the strategies of pre-donation screening to reduce the risk of HBV transmission (9). Iran with a 3% prevalence of HBsAg was previously an intermediate endemic country for HBV infection. Since the adoption of routine HBV neonatal vaccination from 1993, it is estimated that HBV endemicity has decreased, reaching around 1.7% (10). Nevertheless, the reported prevalence of OBI, especially among healthy blood donors, has been fluctuating among various studies in Iran. Part of this difference can depend on the sensitivity and specificity of the assays which have been used in these studies to detect HBV infection. Since a commercial, standardized and valid technique is not available to diagnose OBI, nested PCR method with specified different primers will be useful.

## 2. Objectives

The current study aimed to determine the presence of HBcAb and HBV DNA among Iranian HBsAg negative healthy blood donors who donated their blood to the Tehran Blood Transfusion Center during 2011.

## 3. Patients and Methods

### 3.1. Population

1000 serum samples were obtained from apparently healthy blood donors by the Tehran Blood Transfusion Center offices during November 2011. The inclusion criteria were negative result for HBsAg, HCV antibody and HIV antibody ELISA tests. The sample size formula resulted in 909 samples for the study. Considering an attrition rate of 9% (because of laboratory / genetic examinations), the required sample size will be about 1000 for the study.

### 3.2. Detection of Serologic Markers

HBcAb was screened in the samples by ELISA kit (bioELISA anti HBc, Biokit, Barcelona, Spain).

### 3.3. Detection of Hepatitis B Viral DNA

Following serological tests, DNA was extracted from 200 ul of donor HBcAb positive serum by High Pure viral nucleic acid kit (Roche, Germany). Nested PCR was done on 5 ul of DNA extracted from HBcAb positive samples, 10 ul of BIONEER PCR master mix (BIONEER, S.Korea) and two pairs of primers (25 pmol) which were designed to amplify all genotypes of HBV as described previously by Jazayeri *et al.* (11). HBV genome which was derived from an HBV chronic carrier patient was used as positive control. Distilled water was used as blank control to check material contamination. Lack of contamination was guaranteed by positive and blank controls in each round of nested PCR. For both rounds of nested PCR, DNA samples were denatured at 95°C for 14 min and were subjected to 40 cycles (94°C for 1 min; 55°C for 1 min; 72°C for 90 sec) followed by 72°C for 10 min at final extension in an Eppendorf Thermal Cycler PCR (11). After electrophoresis of PCR products in 1% agarose gel, dyed with ethidium bromide and visualized in UV transilluminator, qualitatively positive samples were reported as OBI cases.

### 3.4. Statistical Analysis

SPSS *vs.* 16 software was employed to analyze the data. Categorized variable were analyzed using Chi Square or Fisher exact tests as appropriate. Numerical variables were analyzed using independent samples t test. Statistical level of significance was considered at *P* < 0.05.

## 4. Results

1000 blood Samples were collected from 64 (6.4%) women and 936 (93.6%) men. The mean ± SD age of the studied population was 37.7 ± 10.5 years. Donation frequency range was 1-92 times (mean: 5.67). 80 positive cases for HBcAb were detected (7 women and 73 men) which were comparable to results from other countries (Table). Male gender was observed in 91% of HBcAb positive donors. The mean ± SD of donation frequency in HBcAb positive donors was 5.9 ± 4.8 and in HBcAb negative donors was 5.7 ± 5.9 (*P* > 0.05). The mean ± SD of age for HBcAb positive donors was 40.4 ± 10.9 years and for HBcAb negative donors was 37.6 ± 10.5 year (*P* > 0.05). HBV viral DNA was detected in 4% of blood donors and in 50% of HBcAb positive donors (1 woman and 39 men). The mean ± SD of age for HBcAb positive donors without HBV DNA was 37.7 ± 10.5 years and for HBcAb positive donors with HBV DNA was 40.9 ± 11.2 years (*P* = 0.05). To the authors` best knowledge, this was the highest percent of HBV DNA reported from HBcAb positive Iranian blood donors in comparison with other Iranian studies.

**Table.** Studies on the Prevalence of HBV-DNA in HBcAb Positive Donated Bloods in Asian Countries and the Middle East

Country	Year	Number of Blood Samples	Serological Test	Molecular Test	HBcAb +, %	Viral DNA+ a, %	OBI b, %	Reference
**South Korea**	2008	12461	ECL immunoassay	multiplex Real time PCR kit	13.5	0.1	0.016	(27)
**India**	2010	12232	ELISA	NA	0.12	NA	NA	(28)
**India**	2012	94247	ELISA	ID NAT	10.22	0.15	0.01	(29)
**Pakistan**	2005	966	ELISA	nested PCR with a commercial kit	17.28	3	0.5	(30)
**Turkey**	2006	1196	ELISA	Pooled Real Time PCR	15	0	0	(31)
**Turkey**	2007	1000	ELISA	Real Time PCR kit	20	0	0	(32)

Abbreviations: NA, not available; ID NAT, individual NAT; ECL, electro chemiluminescence; OBI, occult hepatitis B infection.

^a^Within HBcAb positive patients

^b^Within total population studied

## 5. Discussion

In this study, serum samples of HBsAg negative healthy blood donors were collected from 1000 healthy blood donors and HBcAb of these samples were examined by ELISA assay. 91% of HBcAb positive donors were male and only 9% were female which was not much different from the gender distribution of the blood donor population. A positive relationship was observed between HBcAb positivity and age of the donors and also with HBV DNA positivity. This means that older persons possibly live with HBV for a longer period which can provide an opportunity for the virus to change its genome or set balance with the host immunity and live persistently with its host. Then it can be suggested that HBV infected Iranian patients have been infected in the early decades of their lives. This is in consistent with other studies which had suggested intra familial transmission of HBV as the important way for HBV transmission in Iran (12, 13). The results of this study revealed that half of HBcAb positive samples were also positive for HBV DNA. Therefore a positive relationship between HBcAb positivity and existence of HBV DNA was distinguished. The current research data on HBcAb prevalence were consistent with previous data obtained from other studies in Iran and from other countries in the Middle East. (Table) On the other hand, the relative frequency of patients with positive viral DNA was considerably higher than previous studies in Iran. One important difference was that it was the first time in Iran that HBV DNA in apparently healthy blood donors was detected by nested PCR, while in previous researches single-round PCR was employed to detect viral DNA. It should be emphasized that, at present, the gold standard test for OBI diagnosis is "homemade nested PCR" technique with at least two different sets of primers (7, 14). Single-round PCR or Real time PCR was the only method which was used in previous Iranian studies on OBI among healthy blood donors. Since viral load among OBI people can be low or fluctuating, nested PCR with high sensitivity is a useful technique to detect HBV genome (7, 15). There are some studies about OBI among healthy blood donors in which nested PCR has been used as a critical method to detect HBV genome. Each of these studies has reported a different prevalence of OBI. It seems that HBV endemicity and laboratory methods to detect the infection are the most important factors which lead to these controversial results.

In a case report from Brazil, viral DNA was detected in an HBsAg and HBcAb negative blood donor by nested PCR. The researchers emphasized the importance of nested PCR and recommended NAT to screen donated blood units (16, 17). In Iran, Delavari *et al*. studied 1535 blood donors in Kerman in 2010. They reported that 8 percent of the blood samples negative for HBsAg, were positive for HBcAb. HBV DNA was detected in 29.7% of HBcAb positive samples by Real Time PCR (18). In another study done in Rasht, the prevalence of HBcAb positive samples was 3.8% and only one case was positive for viral DNA (19). In Arak, Sofian *et al*. reported that 2.1% of blood donors were HBcAb positive. They did not detect any viral DNA in the population under study (20). The authors noted that Arak province is a low prevalence region for HBV infection. They did not however provide any proof of this claim. Jafarzadeh *et al.* reported that 5.2% of blood donors in Rafsanjan in 2008 were HBcAb positive and detected HBV DNA in 28.6% of the individuals (21). In Isfahan, the prevalence of HBcAb and viral DNA in HBcAb positive samples were reported 8% and 11.3% respectively (22). Finally in another study in Shiraz done in 2004, 6.5% of blood donors were positive for HBcAb and HBV DNA was detected in 12.2% of the samples (23). Reports from other countries such as Pakistan, South Korea, and Turkey reveal the presence of viral DNA in healthy blood donors with positive HBcAb (Table). The results of studies on the frequency of HBcAb and viral DNA positivity from some Asian countries have been summarized in Table. The differences in results of studies in Iran can be partly explained by different endemicity rates of HBV infection in different regions of Iran. The current study has been done in the capital of Iran which is a mixture of different Iranian ethnicities. The data revealed that the relative frequency of HBcAb and/or DNA positive blood donors has not reduced over this six-year period and has even increased. It seems that more radical changes in screening system of blood donation or HBV vaccination have to be done to solve this old problem in Iran. Studies in Iran on OBI among healthy blood donors suggest that at least HBcAb screening of blood donors may reduce the risk of HBV transmission through transfusion. In spite of the reduction in the rate of HBV infection after vaccination and improvements in the selection criteria for blood donors, transmission of HBV infection by transfusion still occurs specially in developing countries. Blood screening procedures are dependent on HBV endemicity and economic conditions of each country. Some countries like Iran (21) and India (6) only use HBsAg test for blood screening, others like Brazil also use HBcAb ELISA test to ensure blood safety (17). It seems that in countries with high or intermediate prevalence of hepatitis B, HBcAb test may lead to limited blood supplies (6) and it will be better if only HBcAb IgM and not the total HBcAb test is used to screen donated blood units (24). With commercial HBcAb ELISA kits, the total HBcAb (IgM + IgG) is measured. In the case of HBcAb positivity, different scenarios are predicted. One of them is convalescent of hepatitis B disease and another is chronic infection with HBV. If donor’s HBcAb IgM is positive, it means that he is currently infected with HBV and this blood sample is hence infected as well. The common tests which are usually used to detect HBsAg are conventional ELISA and sometimes molecular tests like PCR. In OBI patients, the aforementioned tests have low efficacy because of undetectable HBsAg due to possible viral mutations or imperfect immune responses of the host. Some OBI cases stay uncovered by these assays and have the potential ability to spread viral infection and be a source of HBV transmission through transfusion (25). One of the suggestions of this study is to follow up the people who received donated blood from OBI patients to observe the effect of this donation on their health and evaluate the reactions of their immune system too. More researches should be done in the future to detect and evaluate viral mutations and genetic factors of recipients’ immune system in Iran to clarify the interactions of the virus and the host immune system in different regions with various endemicity rates. And finally as it was confirmed in previous studies in Iran, single round-PCR test is not reliable enough to detect all OBI cases and more sensitive molecular tests such as nested PCR are needed to be done to increase the reliability of the screening. It seems that combination of molecular tests and HBcAb is preferable to HBsAg and HBcAb screening (26). The current study emphasized that OBI was prevalent among 50% of HBcAb positive healthy blood donors. By the "in house nested PCR" more OBI cases can be detected among healthy blood donors in comparison with previous studies in Iran. This matter results in to be more careful about the risk of HBV transmission through transfusion. As Iran was originally an intermediate HBV endemicity rate, which had been reported before HBV vaccination, it seems that HBsAg is not enough to reject infected blood donors. Furthermore, it is highly recommend that HBcAb test can reduce the risk of HBV transmission through transfusion.
